# Müllerian adenosarcoma in a young adult: A case report and literature review of diagnostic and management challenges

**DOI:** 10.1016/j.gore.2026.102069

**Published:** 2026-03-22

**Authors:** Charles Malisaba Posite, Biruk Legesse, Ndungo Etienne, Musubao Justine, Samuel Tumwesigire, Abraham Birungi, Mirna Batista Santos, Suleman Essa Ahmed, Henry Wabinga, Raymond Atwine

**Affiliations:** a Department of Pathology, Kampala International University, Ishaka, Uganda; b Faculty of Medicine, Université Catholique du Graben, Butembo, Democratic Republic of the Congo; c Planetary Health and Climate Resilience Lab, EarthRoost+, Beni, Democratic Republic of the Congo; d Department of Gynecology and Obstetrics, Cliniques Universitaires du Graben, Butembo, Democratic Republic of the Congo; e Department of Pathology, Mbarara University of Science and Technology, Mbarara, Uganda

**Keywords:** MüllerianAdenosarcoma, Young Adult, Fertility Preservation, Recurrence, Case Report, Literature Review

## Abstract

**Background:**

Müllerian adenosarcoma is a rare biphasic uterine malignancy typically affecting postmenopausal women. Its occurrence in young, nulliparous women is extremely uncommon, presenting diagnostic challenges, especially in cases of presumed recurrences.

**Case Presentation:**

A 22-year-old nulliparous woman presented with a presumed recurrent, enlarging prolapsing uterine mass and post-coital bleeding. Her history was notable for two prior excisions of a similar mass without histopathological review. Examination revealed a 6 cm friable mass protruding through the cervical os. Given the recurrence and risk of hemorrhage in a resource-limited setting, a total abdominal hysterectomy was performed. Gross examination identified a 9x6x4 cm polypoid mass confined to the fundus. Histopathology confirmed high-grade endometrial adenosarcoma, characterized by classic biphasic features, significant spindle-cell atypia, and a brisk mitotic rate (>10 mitoses/10 HPFs). Immunohistochemistry showed stromal positivity for CD10 and PR. No myometrial invasion or lymphovascular space invasion was observed. The patient remains asymptomatic under a rigorous three-month surveillance protocol.

**Conclusion:**

This case highlights a rare high-grade presentation in a young adult. Despite aggressive histological features, the absence of myometrial invasion supports definitive surgery followed by intensive long-term surveillance. This report underscores the necessity of a high index of suspicion for malignancy in recurrent polypoid masses within the reproductive age group and addresses critical gaps regarding fertility preservation and age-specific management protocols.

## Highlights

• Clinical presentation often mimics benign polyps, requiring high diagnostic suspicion.
• Diagnosis confirmed by biphasic growth, cambium layer, and CD10/PR positivity.
• Hysterectomy prioritized over fertility preservation due to rapid tumor recurrence.
• Absence of myometrial invasion improves prognosis despite a high mitotic rate.

## 1 Introduction

Müllerian adenosarcoma is a rare, biphasic malignancy of the female genital tract, accounting for approximately 5–10% of all uterine sarcomas (Nathenson et al., 2016, Clement and Scully, 1974). This entity was first comprehensively defined by Clement and Scully in 1974 to characterize the intimate combination of a benign (or mildly atypical) epithelial component and a frankly malignant, sarcomatous mesenchymal stroma (Clement and Scully, 1974). Müllerian adenosarcoma predominantly affects perimenopausal and postmenopausal women, with a mean age of presentation in the sixth decade of life; however, its occurrence in young, premenopausal women, particularly in the context of prior recurrence, presents a diagnostic and therapeutic challenge (Wang et al., 2023, Chen et al., 2024).

The clinical presentation is typically abnormal uterine bleeding or a large, polypoid mass protruding through the external cervical os, mimicking a benign lesion such as an endometrial polyp or leiomyoma. The definitive diagnosis relies on histopathological examination, specifically identifying the characteristic low-power leaf-like or phyllodes-like architecture, the periglandular stromal cuffing (or cambium layer), and malignant mesenchymal elements exhibiting significant cellular atypia (Clement and Scully, 1974, Allen et al., 2018). Crucially, immunohistochemistry (IHC) aids in confirmation, with the malignant stromal component typically demonstrating positive expression for CD10 and progesterone receptor (PR), reflecting endometrial stromal differentiation, while the epithelial component stains positively for cytokeratins (Wang et al., 2023, Nigro et al., 2021).

Management for localised müllerian adenosarcoma is primarily surgical, with total hysterectomy being the gold standard for definitive treatment (El-khalfaoui et al., 2014). The prognosis is governed by the International Federation of Gynaecology and Obstetrics (FIGO) staging system and key pathological factors, notably the presence of myometrial invasion, lympho-vascular space invasion (LVSI), and sarcomatous overgrowth (pure sarcoma comprising >25% of the tumor), which significantly increases the risk of recurrence and mortality (Khan et al., 2023). Given the tumor's propensity for late recurrence, the standard of care for early-stage disease (confined to the endometrium) is complete surgical resection followed by rigorous, long-term surveillance (El-khalfaoui et al., 2014, Khan et al., 2023).

We present the case of a 22-year-old, nulliparous woman with a presumed recurrent uterine adenosarcoma. This report highlights the necessity of a high index of suspicion in young patients and evaluates the management of large, high-mitotic-rate tumors in the context of limited existing evidence for age-specific surveillance.

## 2 Case presentation

A 22-year-old, nulliparous black woman presented at Gynecology Department of Cliniques Universitaires du Graben (CUG), Eastern Democratic Republic of the Congo, with a three-month history of a long-standing, progressively enlarging vaginal mass associated with significant post-coital and contact bleeding. The history was notable for two prior excisions of a similar mass over the preceding 18 months, with unavailable histopathology reports (See Timeline). The patient denied constitutional symptoms, and her history was negative for malignancy. Gynecological history revealed that she was nulliparous who started her menstruation at the age of 12 years with regular menses lasting 3–4 days, which were considered normal. She was not on any contraceptive pill or any other methods of contraception. Gynecological examination revealed a large, fleshy, friable polypoid mass measuring approximately 6 cm. The clinical decision to proceed directly to exploratory laparotomy and subsequent total abdominal hysterectomy with adnexa preservation-without prior cross-sectional imaging or pre-operative biopsy-was predicated on the patient’s history of two previous presumed recurrences within 18 months. Because histopathological review was not performed during the initial surgeries at an outside facility, a definitive pathological link between the masses could not be established; however, the clinical behavior was highly suggestive of a relapsing process. In a resource-constrained setting, given the rapidly enlarging nature of the mass and the risk of life-threatening hemorrhage from the friable tissue, definitive surgical intervention was prioritized to achieve oncological control.

Intraoperatively, the uterus appeared significantly distorted and enlarged; however, this distortion was found to be secondary to a large, soft, intracavitary mass rather than infiltrative growth. The uterine serosa was intact, and no gross evidence of extrauterine extension or adnexal involvement was observed. Given the patient’s age and the macroscopic appearance of localized disease, the ovaries were preserved after thorough inspection.

**Fig. 1:**
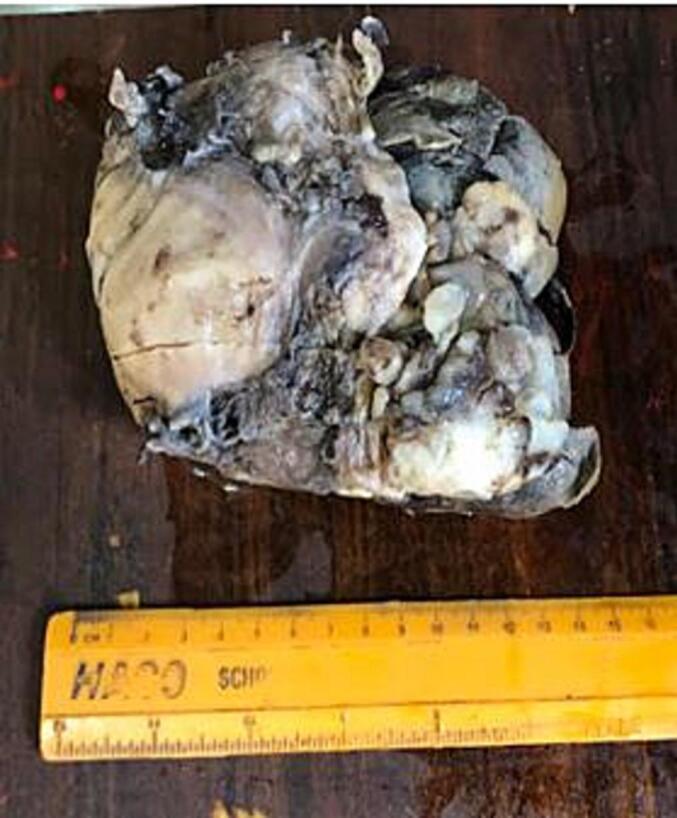
Distorted uterus shows a polypoid, exophytic mass arising from the fundus on a broad base.

**Fig. 2:**
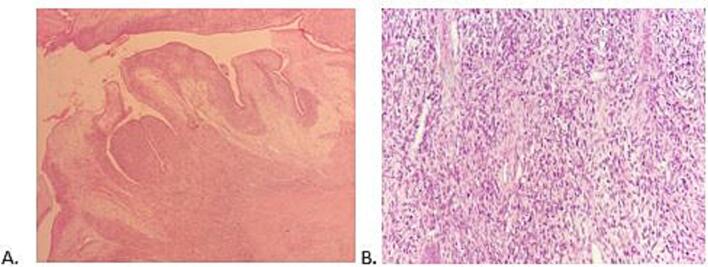
H&E-stained sections show the phyllodes-like architecture (A: x40), benign-appearing, non-atypical endometrial glands, and hypercellular stroma displaying significant spindle-cell atypia, and a brisk mitotic activity (>10 mitoses per 10 HPFs) (C: x200).

**Fig. 3:**
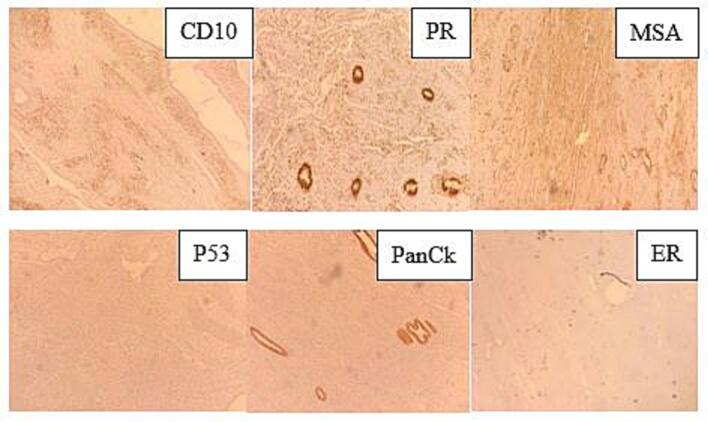
IHC profile. Stromal component: CD10+, PR+, MSA+, p53-, pan-cytokeratin-, and ER-. Epithelial component: pan-cytokeratin+ and PR+.

The uterus contained a 9x6x4 cm polypoid, exophytic mass arising from the fundus on a broad base. It was soft, friable, and confined to the endometrial cavity (Fig. 1). The lesion demonstrated a classic biphasic growth pattern of müllerian adenosarcoma, with phyllodes-like architecture on low power. The epithelial component consisted of glands lined by stratified cuboidal cells with normal mitotic figures consistent with proliferative phase of endometrium. There was mild cystic endometrial glands and no features of atypia. The mesenchymal component was overtly malignant, characterized by high cellularity, significant spindle-cell atypia, and a brisk mitotic activity (>10 mitoses per 10 HPFs), frequently exhibiting periglandular cuffing (Fig. 2). The tumor was strictly confined to the endometrium. Crucially, no myometrial invasion or LVSI was observed. The stromal component was positive for CD10 (confirming endometrial stromal lineage), PR, and muscle-specific actin (MSA); and negative for p53, pan-cytokeratin, and estrogen receptor (ER). The epithelial component was positive for pan-cytokeratin and PR (Fig. 3).

The patient’s postoperative course was uneventful. Given the high-grade features but absence of deep myometrial invasion, she was placed on a rigorous surveillance protocol involving clinical examinations and pelvic imaging every three months for the first year to monitor for recurrence. While pre-operative imaging was bypassed to prioritize emergency surgical control of the friable, bleeding mass, the subsequent histopathological confirmation of malignancy necessitated a shift in resource prioritization. The patient’s follow-up protocol currently utilizes serial pelvic ultrasonography and clinical exams, which are more sustainable in this resource-limited context than advanced cross-sectional imaging (See Table 1.).

**Table 1:** Timeline.

Time	Activity
22 months before the current presentation	Initial onset of the vaginal/uterine mass. Local excision at an outside facility. No histopathology report available.
12 months before the current presentation	First presumed recurrence of the prolapsing uterine mass. Repeat local excision. Histopathology was not performed.
Three months before the current presentation	Onset of a progressively enlarging, prolapsing uterine mass. Significant post-coital and contact bleeding.
January 2025	Patient presents to CUG with a 6 cm fleshy, friable polypoid mass. Clinical diagnosis of presumed recurrent uterine mass; suspected malignancy given the 18-month history of two presumed recurrences.
Day 1 of admission	Pre-operative preparation.
Day 2 of admission	TAH with preservation of adnexa.
Day 1 post-operative	TAH specimen sent for histopathological analysis.
Day 7 post-operative	Patient discharged after an uneventful hospital stay.
One-month post-operative	Histopathology report available with diagnosis of müllerian adenosarcoma.
Follow-up	Clinical examination and pelvic imaging every three months for the first year.

## 3 Discussion

Müllerian adenosarcoma is an infrequent uterine malignancy, and its presentation in a nulliparous 22-year-old is exceptionally rare, contrasting sharply with the typical postmenopausal demographic (Nathenson et al., 2016, Wang et al., 2023). In postmenopausal women several factors have been thought to play a role in the pathogenesis of this tumor, including oestrogenism, pelvic irradiation, and tamoxifen use for breast cancer. In the young women, there appear to be no specific factors, which could be thought to play a role in the pathogenesis of this tumor. However, we note that this is a nulliparous woman who had an early menarche of 12 years suggesting that she has been exposed to prolonged estrogen. In a study of Clement and Scully nulliparity was found in 15 out of 49 cases suggesting hormonal imbalance could play a role (Clement and Scully, 1974). Müllerian adenosarcoma can be clinically mistaken for common benign entities like complex endometrial polyps or prolapsed leiomyomas, especially when only superficial fragments are removed, which may lack the diagnostic malignant stromal component (Angelo and Prat, 2010). This case emphasizes the urgent need for a high index of suspicion and complete sampling of any recurrent, friable polypoid uterine mass in reproductive-age women.

The definitive diagnosis of müllerian adenosarcoma hinges on the characteristic biphasic growth pattern observed microscopically, where benign-to-mildly-atypical glands are surrounded by a malignant mesenchymal stroma (Clement and Scully, 1974, Allen et al., 2018). Our case demonstrated this classic pattern with the stromal component exhibiting marked cellular atypia and a brisk mitotic activity of >10 mitoses per 10 HPFs. While the World Health Organization (WHO) definition requires only 2 mitoses/10 HPFs in the stroma, a rate of 5 mitoses/10 HPFs is commonly associated with an increased risk of recurrence (Seagle et al., 2016, Li et al., 2022).

IHC was vital in confirming the lineage, showing CD10 positivity in the stromal cells, a strong marker of endometrial stromal differentiation, alongside positivity for PR and MSA, consistent with the tumor's müllerian origin (Nigro et al., 2021, Li et al., 2022). The clinical size of the tumor 9 cm is also a significant concern, as large size has been independently suggested as a poor prognostic factor, potentially correlating with a higher risk of recurrence (Khan et al., 2023).

The current standard of care for localized uterine müllerian adenosarcoma is total hysterectomy (El-khalfaoui et al., 2014). Our patient was diagnosed with the tumor strictly confined to the endometrium with no myometrial invasion, a critical favorable prognostic factor (Khan et al., 2023). The decision to perform a hysterectomy while preserving the adnexa was appropriate given the patient's young age and nulliparity, as müllerian adenosarcoma rarely metastasizes to the ovaries, especially in early-stage disease without deep myometrial invasion or sarcomatous overgrowth (Wang et al., 2023, El-khalfaoui et al., 2014).

While the high mitotic rate and large tumor size are traditionally poor prognostic indicators, the clinical outlook is partially mitigated by the absence of sarcomatous overgrowth and deep myometrial invasion, both of which are independent predictors of recurrence and metastasis in uterine adenosarcoma. However, müllerian adenosarcoma carries a significant risk of recurrence (15–25%), usually locoregional and often occurring late, sometimes years after initial surgery (Chen et al., 2024, Khan et al., 2023). Given the tumor's high-grade features (mitotic rate > 10 HPFs), this patient faces an elevated risk profile compared to an average low-grade case.

Therefore, the recommended management plan, which involves no immediate adjuvant therapy but necessitates rigorous, long-term clinical surveillance (initial three-month intervals for one year), aligns with current guidelines for high-risk Stage I disease, where the benefit of adjuvant radiotherapy or chemotherapy has not been definitively proven to improve overall survival (El-khalfaoui et al., 2014, Khan et al., 2023). Long-term follow-up remains paramount due to the potential for late recurrence or progression.

In young women, there appear to be no specific factors in the pathogenesis; however, prolonged estrogen exposure from early menarche may be relevant. Clinicians should also consider screening for familial syndromes, such as DICER1-related pleuropulmonary blastoma syndrome, which has been associated with uterine adenosarcomas in children and young adults (Höhn et al., 2021). Due to limited resources, our patient was not screened for this.

A primary challenge in this demographic is the balance between oncological safety and fertility preservation. While total hysterectomy remains the gold standard, some guidelines suggest that for Stage IA tumors without sarcomatous overgrowth, uterine-preserving surgery (polypectomy or local excision) may be considered, provided the patient accepts a higher risk of local recurrence and commits to ultra-close monitoring (Nigro et al., 2021). In this case, the high mitotic rate and recurrence history made hysterectomy the safer clinical choice.

A review of the SARCUT study and recent SEER database analyses reveals that while the median age for uterine adenosarcoma is 58 years, only a fraction of cases occur under the age of 30 (Seagle et al., 2016, Mancari et al., 2024). Most reported cases in young women are low-grade; however, our patient’s tumor exhibited a mitotic rate of >10/10 HPFs. In the literature, high mitotic activity is typically clustered with sarcomatous overgrowth (SO) and deep invasion. Our case is an outlier: a high-grade stromal component confined strictly to the endometrium. This suggests that mitotic rate alone, in the absence of SO, may not necessitate the aggressive adjuvant therapies often reserved for high-grade sarcomas, even in young patients where recurrence-free survival is prioritized.

Limitations of this report include the lack of preoperative MRI/CT imaging to assess baseline pelvic status and the relatively brief follow-up period. Long-term surveillance must be multifaceted, incorporating clinical exams, imaging (ultrasound or MRI), and laboratory assessments to detect late-stage recurrence, which is a known characteristic of this malignancy (Wang et al., 2023, Mancari et al., 2024).

## 4 Conclusion

This case report highlights the rare occurrence of a high-grade endometrial adenosarcoma in a 22-year-old, nulliparous woman, emphasizing the challenge of diagnosing this malignancy in its recurrent, polypoid form. Despite aggressive pathological features, the lack of myometrial invasion supported definitive surgical resection. This report reinforces the need for meticulous histopathology and highlights the necessity for standardized management protocols for müllerian adenosarcoma in the reproductive-age population.

## CRediT authorship contribution statement

**Charles Malisaba Posite:** Writing – review & editing, Writing – original draft, Validation, Data curation, Conceptualization. **Biruk Legesse:** Writing – review & editing, Validation. **Ndungo Etienne:** Writing – review & editing, Validation, Supervision, Data curation. **Musubao Justine:** Writing – review & editing, Validation, Supervision, Data curation. **Samuel Tumwesigire:** Writing – review & editing, Validation. **Abraham Birungi:** Writing – review & editing, Validation, Data curation. **Mirna Batista Santos:** Writing – review & editing, Validation, Supervision, Data curation. **Suleman Essa Ahmed:** Writing – review & editing. **Henry Wabinga:** Writing – review & editing, Validation, Supervision, Data curation. **Raymond Atwine:** Writing – review & editing, Validation, Supervision, Data curation.

## Ethics approval

The work was conducted in accordance with the Declaration of Helsinki. Institutional ethical approval was not required to publish the case details.

***Consent for publication***

Written informed consent was obtained from the patient for publication of this case report and any accompanying images. A copy of the written consent is available for review by the Editor-in-Chief of this journal on request.

***Declaration of generative AI and AI-assisted technologies in the manuscript preparation process***.

During the preparation of this work, the authors used Grammarly in order to improve the quality, clarity, and effectiveness of written English. After using this tool, the authors reviewed and edited the content as needed and take full responsibility for the content of the published article.

## Funding

The authors declare that no funds, grants, or other support were received during the preparation of this manuscript.

## Declaration of competing interest

The authors declare that they have no known competing financial interests or personal relationships that could have appeared to influence the work reported in this paper.
